# Role of Fibroblast Growth Factor 23 (FGF23) and αKlotho in Cancer

**DOI:** 10.3389/fcell.2020.601006

**Published:** 2021-01-14

**Authors:** Franz Ewendt, Martina Feger, Michael Föller

**Affiliations:** ^1^Department of Nutritional Physiology, Institute of Agricultural and Nutritional Sciences, Martin-Luther University Halle-Wittenberg, Halle, Germany; ^2^Department of Physiology, University of Hohenheim, Stuttgart, Germany

**Keywords:** Ca^2+^, calcitriol, inflammation, malignancies, phosphate

## Abstract

Together with fibroblast growth factors (FGFs) 19 and 21, FGF23 is an endocrine member of the family of FGFs. Mainly secreted by bone cells, FGF23 acts as a hormone on the kidney, stimulating phosphate excretion and suppressing formation of 1,25(OH)_2_D_3_, active vitamin D. These effects are dependent on transmembrane protein αKlotho, which enhances the binding affinity of FGF23 for FGF receptors (FGFR). Locally produced FGF23 in other tissues including liver or heart exerts further paracrine effects without involvement of αKlotho. Soluble Klotho (sKL) is an endocrine factor that is cleaved off of transmembrane Klotho or generated by alternative splicing and regulates membrane channels, transporters, and intracellular signaling including insulin growth factor 1 (IGF-1) and Wnt pathways, signaling cascades highly relevant for tumor progression. In mice, lack of FGF23 or αKlotho results in derangement of phosphate metabolism and a syndrome of rapid aging with abnormalities affecting most organs and a very short life span. Conversely, overexpression of anti-aging factor αKlotho results in a profound elongation of life span. Accumulating evidence suggests a major role of αKlotho as a tumor suppressor, at least in part by inhibiting IGF-1 and Wnt/β-catenin signaling. Hence, in many malignancies, higher αKlotho expression or activity is associated with a more favorable outcome. Moreover, also FGF23 and phosphate have been revealed to be factors relevant in cancer. FGF23 is particularly significant for those forms of cancer primarily affecting bone (e.g., multiple myeloma) or characterized by bone metastasis. This review summarizes the current knowledge of the significance of FGF23 and αKlotho for tumor cell signaling, biology, and clinically relevant parameters in different forms of cancer.

## Fibroblast Growth Factor 23 (FGF23)

The human *fibroblast growth factor 23* (*FGF23*) gene localized on chromosome 12p13 was discovered in 2000 (*Autosomal dominant hypophosphataemic rickets is associated with mutations in FGF23*, 2000, ADHR Consortium, 2000). FGF23 is a member of the family of fibroblast growth factors (FGFs) and a proteohormone of 32 kDa (Yamashita et al., 2000; Yamazaki et al., 2002). It is characterized by endocrine and paracrine effects in contrast to most other FGFs, which do not act as classical hormones (Angelin et al., 2012). Endocrine FGF23 is primarily produced by bone cells and released into the bloodstream (Riminucci et al., 2003; Yoshiko et al., 2007). Low *Fgf23* expression was detected in other tissues, such as spleen, thymus, small intestine, liver, kidney, heart, and brain (Yamashita et al., 2000; Yoshiko et al., 2007). The secretion of the biologically active hormone into the blood is controlled by proteolytic cleavage of the full-length, intact FGF23 molecule by a furin/furin-like proprotein convertase between ^179^Arg and ^180^Ser (Shimada et al., 2001). The susceptibility of FGF23 to proteolytic degradation is regulated by UDP-N-acetyl-alpha-D galactosamine: polypeptide N-acetylgalactosaminyltransferase 3 (GalNT3)-mediated *O*-glycosylation at threonine 178 and phosphorylation at serine 180 by the enzyme family with sequence similarity 20 member C (FAM20C) (Tagliabracci et al., 2014). Target organs of FGF23 include kidney and parathyroid glands (Ben-Dov et al., 2007; Gattineni et al., 2009). In the former, FGF23 inhibits the reabsorption of phosphate by down-regulating the membrane abundance of NaPiIIa, the major Na^+^-coupled phosphate transporter of the proximal tubule (Gattineni et al., 2009). Moreover, FGF23 suppresses the synthesis of 1,25(OH)_2_D_3_, active vitamin D, by inhibiting key enzyme 1-α-hydroxylase (encoded by *Cyp27b1*) in the kidney (Chanakul et al., 2013). In the parathyroid glands, FGF23 down-regulates the production and secretion of parathyroid hormone (PTH) (Ben-Dov et al., 2007). This way, FGF23 is part of a hormone circuit additionally involving PTH and 1,25(OH)_2_D_3_ and regulating phosphate and vitamin D metabolism, as well as impacting on Ca^2+^ (Blau and Collins, 2015). These endocrine effects of FGF23 are mediated by FGF receptors (FGFRs) including FGFR1c, FGFR3c, and FGFR4 with αKlotho (KL) serving as a scaffolding protein, which is needed to enhance the binding affinity of FGF23 (Gattineni et al., 2009, 2011; Chen G. et al., 2018). Other effects of locally produced FGF23 are, at least in part, paracrine and include the regulation of inflammation in hepatocytes (Singh et al., 2016), the induction of cardiac hypertrophy (Faul et al., 2011), or inhibition of neutrophils (Rossaint et al., 2016). At least some of these effects are independent of KL (Quarles, 2019). The plasma concentration of FGF23 goes up in many acute and chronic diseases (Gutierrez et al., 2005). In chronic kidney disease (CKD), high FGF23 plasma levels are observed prior to hyperparathyroidism or hyperphosphatemia (Isakova et al., 2011). FGF23 predicts progression and outcome in CKD (Hasegawa et al., 2010). Independently of kidney disease, FGF23 is associated with carotid atherosclerosis (Rodríguez-Ortiz et al., 2020), fibrosis, and poorer prognosis in heart failure (Roy et al., 2020) and prognosis in heart failure with preserved ejection fraction (Kanagala et al., 2020). In another cohort, however, the role of FGF23 for patients with heart failure was less clear (Stöhr et al., 2020). Dyslipidemia is associated with higher FGF23 levels (Mirza et al., 2011). Inflammatory conditions also up-regulate FGF23 (Czaya and Faul, 2019). Hence, FGF23 is discussed as a biomarker correlating with progression and outcome in some significant diseases of high burden (Schnedl et al., 2015).

## αKlotho

**FIGURE 1:**
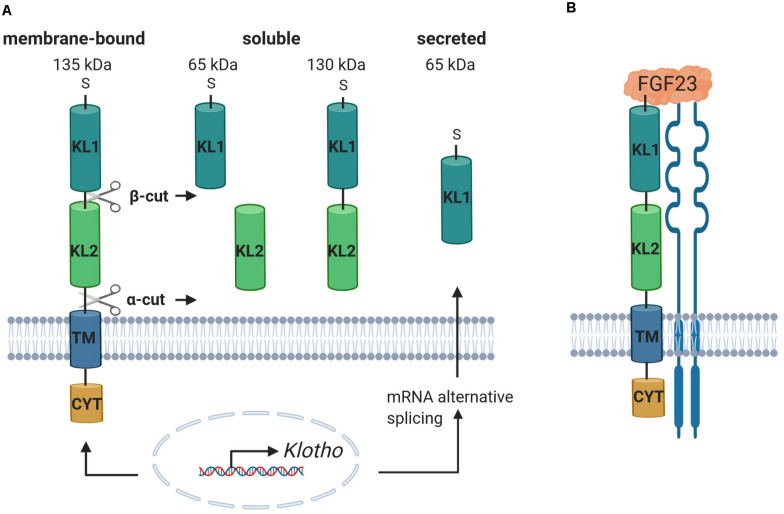
Klotho includes different isoforms and binds to FGFR, facilitating the binding and subsequent signal transduction of FGF23. **(A)** The *KL* gene encodes a single-pass transmembrane protein, comprising an N-terminal short signal sequence (S), the ectodomain, containing the internal repeats KL1 and KL2, the transmembrane domain (TM), and a short cytoplasmic domain (CYT). It exists as full-length 135-kDa membrane-bound KL, the 130-kDa shed soluble KL (sKL) isoform, and the truncated secreted variant of KL. Proteolytic cleavage of full-length KL due to α-cut and/or β-cut produces sKL, containing either KL1, KL2, or both. Alternative RNA splicing of *KL* mRNA generates the secreted isoform, containing an N-terminal signal sequence and KL1 only. **(B)** The complexation of KL with FGFR enables the binding of FGF23, resulting in the formation of a trimeric complex, which activates the downstream signaling pathway. Created with BioRender.com.

The α*Klotho* (referred to as KL) gene was identified in 1997. In mice, a mutation of the *Kl* gene causes a syndrome of rapid aging including a drastically shortened life span and further age-associated diseases and symptoms affecting most organs and tissues such as atherosclerosis, osteoporosis, skin atrophy, infertility, or emphysema (Kuro-O et al., 1997). KL is mainly expressed in the kidney but also in the central nervous system (cerebellum, cerebral cortex, spinal cord) and in other tissues with detectable but lower expression such as thyroid gland, aorta, urinary bladder, ovary, skeletal muscle, pancreas, prostate gland, testis, or the adrenal gland (Kuro-O et al., 1997; Lim et al., 2015). However, it has not been clear for a long time how KL develops its function until it was discovered that the phenotype of the *Kl* knockout mouse is similar to the *Fgf23* knockout mouse. The mice exhibit high serum phosphate levels, soft tissue and vascular calcification, increased expression of renal sodium phosphate cotransporter NaPiIIa, and 1-α-hydroxylase, accompanied by high serum levels of 1,25(OH)_2_D_3_ (Tsujikawa et al., 2003; Nakatani et al., 2009; Razzaque, 2009a). Moreover, it could be shown that the ablation of 1,25(OH)_2_D_3_ signaling in mice lacking a functional vitamin D receptor prevents the premature aging phenotype in *Kl*^–/–^ mice (Anour et al., 2012; Andrukhova et al., 2017). Deficiency of both *1-*α*-hydroxylase* and *Kl* prevents soft tissue and vascular calcification and normalizes the high Fgf23 and low PTH levels paralleled by Kl deficiency in mice (Ohnishi et al., 2009). These findings assign KL an important physiological role in the regulation and maintenance of phosphate homeostasis (Razzaque, 2009b). The human *KL* gene is located on chromosome 13q12 and ranges over 50 kb with 5 exons and 4 introns (Matsumura et al., 1998). It encodes the KL protein, which shows homology with family I β-glycosidases and is a 135-kDa single-pass transmembrane protein (Kuro-O et al., 1997; Chen et al., 2007; Xu and Sun, 2015; Dalton et al., 2017). The protein comprises a N-terminal short signal sequence, the large ectodomain containing two internal repeats termed KL1 and KL2 mediating KL activity and function, the transmembrane domain, and a short intracellular domain (Kuro-O et al., 1997; Kuro-O, 2008; Xu and Sun, 2015) (Figure 1). Three different isoforms can be distinguished: full-length transmembrane KL, the 130-kDa shed soluble form (sKL), and the shorter truncated secreted variant of KL (65 kDa) (Kuro-O et al., 1997; Shiraki-Iida et al., 1998; Dalton et al., 2017). sKL consists of the KL1 and KL2 domain but lacks the transmembrane and intracellular domain. It arises because of proteolytic cleavage, termed α-cut, of full-length transmembrane KL on the cell surface by α-secretases A disintegrin and metalloproteinase domain-containing proteins 10 and 17 (ADAM10 and ADAM 17) and the β-APP cleaving enzyme 1 (BACE1) (Chen et al., 2007; Bloch et al., 2009; Xu and Sun, 2015). The residual transmembrane fragment undergoes an intramembrane proteolytic degradation by γ-secretases (Bloch et al., 2009). Moreover, another cleavage mechanism of KL by ADAM10 and ADAM17, termed β-cut, generates the two 65-kDa fragments KL1 and KL2 (Chen et al., 2007). Therefore, after shedding, sKL protein enters blood, urine, or cerebrospinal fluid as KL1 or KL2 only or both KL1 and KL2 and exerts its functions in other tissues and organs (Imura et al., 2004; Akimoto et al., 2012; Xu and Sun, 2015; Dalton et al., 2017). sKL inhibits insulin growth factor 1 receptor (IGF-1R)/phosphoinositide 3-kinase (PI3K)/AKT serine/threonine kinase (AKT) signaling and activates forkhead box O (FOXO) (Kurosu et al., 2005; Yamamoto et al., 2005). It increases glucose uptake and glycogen storage and reduces lipid accumulation and insulin resistance through PPARα expression (Gu et al., 2020) corroborating the role of KL and underlying signaling in glucose metabolism and adipocyte maturation as discussed recently (Razzaque, 2012). Other KL downstream effects are the activation of extracellular signal–related kinase 1/2 (ERK1/2) (Maekawa et al., 2011), inhibition of Wnt signaling (Liu et al., 2007), or reduction of inflammation (Maekawa et al., 2009). Moreover, sKL is involved in the stimulation of ion channels and transporters including transient receptor potential ion channel TRPV5 (Chang et al., 2005; Cha et al., 2008) or renal outer medullary potassium channel 1 (ROMK1) (Cha et al., 2009). The secreted isoform of KL is processed by alternative RNA splicing in the internal splice donor site of exon 3, containing a N-terminal signal sequence and KL1 only (Matsumura et al., 1998). In contrast to KL and sKL, the secreted isoform has not been detected *in vivo* yet (Kuro-O, 2019).

Progressing CKD is associated with decreased renal KL expression and loss of renal function (Koh et al., 2001; Komaba et al., 2010; Hu et al., 2011). Lower KL expression correlates with more cardiovascular events in patients on hemodialysis (Memmos et al., 2019). In addition, KL inhibits inflammation (Maekawa et al., 2009; Zhao et al., 2011) and oxidative stress (Kimura et al., 2018; Qian et al., 2018), conditions enhanced in CKD (Mihai et al., 2018) and cardiovascular diseases (Dhiman et al., 2015).

In addition, the KL family includes two other members, termed βKlotho and γKlotho (referred to as KLB and KLG hereinafter). The *Klb* gene, identified in 2000, shows sequence similarity to *Kl* and encodes a single-pass transmembrane protein (Ito et al., 2000). KLB is localized in the cell membrane and mainly expressed in the liver and adipose tissue, where it forms a complex with FGFR1 and FGFR4, and mediates metabolic functions of FGF19 and FGF21 (Kurosu et al., 2007; Ogawa et al., 2007; Xu and Sun, 2015). FGF19 controls bile acid synthesis through suppression of *Cyp7a1* (Kurosu et al., 2007). Thus, *Klb*^–/–^, *Fgf15*^–/–^, and also *Fgfr4*^–/–^ mice lack Cyp7a1 suppression, resulting in increased bile acid production and excretion (Inagaki et al., 2005; Ito et al., 2005). Moreover, KLB is necessary for FGF21 signaling, which is expressed mainly in the liver, where it is involved as downstream target of peroxisome proliferator–activated receptor α (PPARα) in metabolic adaptation to fasting but also in adipose tissue, where it modulates lipolysis and glucose uptake (Kurosu et al., 2007; Arner et al., 2008; Suzuki et al., 2008; Dolegowska et al., 2019). The *Klph* gene was found in mice, encoding the Klotho lactase-phlorizin hydrolase-related protein, which is mainly expressed in the eyes but also in the kidney, adipose tissue, and skin (Ito et al., 2002; Fon Tacer et al., 2010). This novel member of the KL family is also termed KLG. KLG interacts with FGFR1b, 1c, 2c, and 4 and promotes activation of FGF signaling by FGF19 in HEK293 cells (Fon Tacer et al., 2010).

## FGF23 and Cancer

As detailed below and summarized in Table 1, the implications of FGF23 in cancer biology are thus far sparser than the known role of its coreceptor KL in tumor diseases. This may, in large part, be due to the fact that KL acts as a tumor suppressor in various types of cancer, whereas such a function is not established for FGF23. A role of FGF23 in malignancies is most clearly proven in the case of tumor-induced osteomalacia (TIO) or oncogenic hypophosphatemic osteomalacia (Larsson et al., 2003). This is a rare paraneoplastic syndrome due to a tumor excessively producing FGF23, which, in line with its main endocrine effects, induces renal phosphate excretion, as well as reduction of 1,25(OH)_2_D_3_. As a consequence of both, the patients suffer from osteomalacia, demineralized bone (Yamazaki et al., 2002; Larsson et al., 2003). Benign soft tissue (mesenchymal) tumors are most frequently responsible for TIO (Boland et al., 2018), but also malignancies including colon adenocarcinoma (Leaf et al., 2013), ovarian cancer (Lin et al., 2014), small cell carcinoma of the lung (Sauder et al., 2016), anaplastic thyroid carcinoma (Abate et al., 2016), B-cell non-Hodgkin lymphoma (Elderman et al., 2016), breast cancer (Savva et al., 2019), and intracranial tumors (Colazo et al., 2020) can produce FGF23. If the causative tumor cannot be identified, the anti-FGF23 monoclonal antibody KRN23 may be therapeutically useful in TIO (Minisola et al., 2017).

**TABLE 1:** Associations of FGF23 with cancer.

Cancer	FGF23 level	FGF23 effect	References
Tumor-induced osteomalacia	↑ Cancer tissues ↑ Serum	↑ Renal phosphate wasting ↓1,25(OH)_2_D_3_ → Osteomalacia	Yamazaki et al., 2002; Larsson et al., 2003; Leaf et al., 2013; Lin et al., 2014; Abate et al., 2016; Elderman et al., 2016; Sauder et al., 2016; Boland et al., 2018; Savva et al., 2019; Colazo et al., 2020
Bone metastasis	↑ Serum		Mansinho et al., 2019
Myelodysplastic syndromes	↑ Serum ↑ Erythroid precursors	↓ Bone mineralization; microarchitecture ↑ *Alpl*; *Runx2* ↑ Anemia	Weidner et al., 2020
Multiple myeloma	↑ Serum ↓ Cells	↑ *EGR-1* and *HPSE* → Impacts tumor growth	Suvannasankha et al., 2015
Prostate cancer	Expression in cells ↔ Serum	↑ Cell proliferation and tumor invasion ↑ MAPK and AKT → Impacts tumor growth	Lee et al., 2014; Feng et al., 2015; Vlot et al., 2018
Endometrial cancer	↔ Serum		Cymbaluk-Płoska et al., 2020
Ovarian cancer	↑ Serum ↑ Cells		Tebben et al., 2005
Colorectal cancer	Serum level may rise ↑ Stool		Jacobs et al., 2011; Wang H.-P. et al., 2014
Breast cancer	↑ Cell mRNA		Aukes et al., 2017
Urothelial carcinoma	↑ Serum		Li et al., 2019
Prolactinoma	↔ Serum		Arslan et al., 2017

### Hematologic Malignancies

Because bone is the main site of FGF23 production, malignancies typically affecting or arising from bone may have a link to FGF23. In patients with bone metastasis due to different solid tumors, a higher FGF23 plasma concentration is associated with shorter survival and shorter time to skeletal-related events (Mansinho et al., 2019). Patients with myelodysplastic syndrome (MDS) characterized by impaired hematopoiesis in the bone marrow have a higher FGF23 plasma concentration that is associated with anemia and lower bone mineralization (Weidner et al., 2020). In mice, MDS is paralleled by *Fgf23* expression in erythroid precursor cells (Weidner et al., 2020). Multiple myeloma (MM) is characterized by painful bone lesions. MM cells exhibit KL-dependent FGF23 signaling, and intact FGF23 plasma levels are elevated in MM patients (Suvannasankha et al., 2015).

### Prostate Cancer

*FGF23* single-nucleotide polymorphisms (SNPs) are associated with increased risk of prostate cancer (Kim et al., 2014a). *FGF23* expression is enhanced in patients with castration-resistant prostate cancer, as well as FGF23/FGFR1/KL in different prostate cancer cell lines (Lee et al., 2014). FGF23 acts as an autocrine factor in prostate cancer cells stimulating tumor invasion and cell proliferation (Feng et al., 2015). According to another study, *KL* expression is reduced due to promoter hypermethylation (Seo et al., 2017). FGF23 down-regulation suppresses tumor growth *in vivo* (Feng et al., 2015). FGF23 production may be subject to autocrine stimulation through FGFR in prostate cancer (Feng et al., 2012; Wu et al., 2013; Lee et al., 2014). According to one study, the FGF23 plasma level is unchanged in prostate cancer (Vlot et al., 2018), although prostate cancer cells may stimulate FGF23 expression in osteocytes (Choudhary et al., 2018). Bone metastasis may account for the high FGF23 levels and symptoms of TIO observed in patients with prostate cancer according to other studies (Nakahama et al., 1995; Cotant and Rao, 2007; Chiam et al., 2013).

### Gynecologic Tumors

In endometrial cancer, no change in the FGF23 plasma concentration is observed (Cymbaluk-Płoska et al., 2020), whereas the FGF23 plasma concentration goes up in advanced-stage epithelial ovarian cancer (EOC) (Tebben et al., 2005), and a defined FGF23 SNP is associated with better prognosis in this tumor entity (Meng et al., 2014). Breast cancer may be associated with oncogenic osteomalacia and raised FGF23 levels (Savva et al., 2019). *FGF23* mRNA expression is high in breast cancer cells, and FGF produced by tumor cells contributes to metastatic lesions (Aukes et al., 2017). Furthermore, FGFR signaling may be highly relevant for breast cancer oncogenesis (Navid et al., 2020). According to a phase 0/1 clinical trial, combined aromatase and FGFR1 inhibition in breast cancer results in a surge in the FGF23 plasma concentration (Quintela-Fandino et al., 2019).

## FGF23 in Other Forms of Cancer

The plasma FGF23 concentration may rise in colorectal adenoma (Jacobs et al., 2011), and FGF23 excretion is enhanced in the stool from patients with colorectal carcinoma (Wang H.-P. et al., 2014). In urothelial carcinoma, an increase in the FGF23 plasma concentration is reported (Li et al., 2019). In patients with prolactinoma, the FGF23 plasma concentration is unaltered, and there is only a minor effect of FGF23 on bone loss in these patients, if any (Arslan et al., 2017). Progression of hepatocellular carcinoma (HCC) is not linked to altered FGF23 expression (Zou et al., 2018).

It is important to keep in mind that most of the aforementioned studies on FGF23 and different types of cancer report associations, not necessarily causative relationships.

## αKlotho Signaling Pathways Relevant for Cancer

The development of cancer, its progression, and metastasis are a complex process. Initially, cells are exposed to harmful genetic or epigenetic alterations resulting in dysregulated signaling pathways. Subsequently, the modified cells escape homeostatic checks and elimination (Sever and Brugge, 2015). Typical dysregulated pathways in cancer include IGF-1R, PI3K/AKT1/mammalian target of rapamycin (mTOR), mitogen-activated protein kinase (MAPK)/ERK, glycogen synthase kinase-3β (GSK-3β), or Wnt/β-catenin signaling. Many of them are controlled by KL (Sopjani et al., 2015; Badve and Kumar, 2019). Moreover, aging is a major driver of cancer (Aunan et al., 2017). Also in view of rapid aging of Kl-deficient mice (Kuro-O et al., 1997), it is intriguing to speculate that KL signaling in many tissues is implicated in cancer development and may be a possible target in cancer prevention or therapy. The role of KL in different forms of cancer is summarized in Table 2.

**TABLE 2:** Associations of KL with cancer.

Cancer	KL level	Mechanism of KL change	KL effect	References
Breast cancer	↓ Cancer tissues ↓ Cell lines	Epigenetic silencing; *KL* variant	↓ Cell proliferation ⊣ IGF-1R/AKT/GSK-3β and ERK1/2 ↑ FGF pathway → Regulation of endoplasmic reticulum Ca^2+^ storage	Wolf et al., 2008, 2010; Rubinek et al., 2012; Dallol et al., 2015; Sinha et al., 2015; Shmulevich et al., 2020
Colorectal cancer	↓ Cancer tissues ↓ Cell lines	Epigenetic silencing; *miR-15b*; NF-κB and IGF-1R activity; *KL* variant	↓ Cell survival; proliferation ↓ Tumor growth; weight; volume ↑ Cell cycle arrest ↑ Apoptosis ⊣ IGF-1R/PI3K/AKT ⊣ ERK and HIF-1α ⊣ NF-κB ⊣ Wnt3a/β-catenin signaling	Gan et al., 2011; Pan et al., 2011; Li et al., 2014, 2016, 2018; Yang et al., 2014; Bordonaro and Lazarova, 2015; Perveez et al., 2015; Arbel Rubinstein et al., 2019; Liu et al., 2019; Xie et al., 2019, 2020; Kamal et al., 2020; Son et al., 2020
Lung cancer	↓ Cancer tissues ↓ Cell lines	*miR-10b*; Ras8 activity	↓ Cell proliferation; growth; invasiveness; migration ↑ Apoptosis ⊣ IGF-1R/AKT ⊣ Wnt3a/β-catenin signaling ↓ IL-6 and IL-8 → Sensitizes for cisplatin via PI3K/AKT or autophagy	Chen et al., 2010, 2012, 2016, 2019; Wang X. et al., 2011, Wang et al., 2013; Huang et al., 2015; Chen B. et al., 2018
Hepatocellular cancer	↓ Cancer tissues ↓ Cell lines	Epigenetic silencing	↓ Colony formation; proliferation; migration; invasion ↑ Apoptosis; autophagy ⊣ Wnt/β-catenin signaling ⊣ IGF-1R/AKT/ERK ↑ VEGFR2/PAK1 →↑ migration; invasion	Chen et al., 2013; Shu et al., 2013; Xie et al., 2013b; Sun et al., 2015; Tang et al., 2016b
Squamous cell carcinoma	↓ Cancer tissues	Epigenetic silencing	⊣ *N*-cadherin → Regulation of EMT	Adhikari et al., 2017; Ibi et al., 2017
Pancreatic cancer	↓ Cancer tissues ↓ Cell lines	Epigenetic silencing; *miR-199a*	↓ Colony size and number; tumor growth ↑ Chemotherapeutic effects ⊣ IGF-1R/AKT/ERK1/2 ⊣ mTOR ⊣ FGF2	Abramovitz et al., 2011; Jiang et al., 2014; Zhang et al., 2020
Gastric carcinoma	↓ Cancer tissues ↓ Cell lines	Epigenetic silencing; *miR-199a*	↓ Growth ⊣ IGF-1R/PI3K/mTOR ⊣ ERK1/2 ↑ Apoptosis	Wang L. et al., 2011; Xie et al., 2013a; He et al., 2014
Prostate cancer	↓ Cell lines	Epigenetic silencing; *KL* SNP		Kim et al., 2014b; Seo et al., 2017
Renal cell carcinoma	↓ Cancer tissues ↓ Cell lines		↓ Cell proliferation; migration; invasion; motility; EMT ⊣ IGF-1R ⊣ PI3K/AKT/GSK-3β/Snail ⊣ EGF-1 dependent p38MAPK activation	Zhu et al., 2013; Gigante et al., 2015; Kim et al., 2016; Dehghani et al., 2018
Ovarian cancer	↓ Cancer tissues ↓ Cell lines		↓ Cell proliferation ↓ Tumor growth and tumor-associated inflammation ⊣ IGF-1/ERK1/2	Lojkin et al., 2015; Yan et al., 2017
Melanoma	↓ Aged cells	PPARγ; HMGB1 and NF-κB activity	↓ Cell motility ⊣ Wnt5a-mediated filamin A cleavage	Camilli et al., 2011; Xie et al., 2016; Behera et al., 2017
Thyroid cancer	↓ Cancer tissues		↓ Cell proliferation ↑ Apoptosis ⊣ Stanniocalcin-1	Dai et al., 2016; Pawlikowski et al., 2019
Urothelial carcinoma of the bladder	↓ High- grade cancer tissues			Hori et al., 2016
Glioblastoma multiforme		Epigenetic silencing	↓ Cell viability	Peshes-Yeloz et al., 2019
Cervical carcinoma	↓ Cancer tissues ↓ Cell lines	Epigenetic silencing	↓ EMT ⊣ Wnt/β-catenin signaling	Lee et al., 2010; Chang et al., 2012
Dedifferentiated liposarcoma	↓ Cancer tissues		↓ Cell proliferation ↑ Apoptosis → Sensitizes to ER stress ⊣ IGF-1–induced Ca^2+^ and ERK1/2 signaling	Delcroix et al., 2018
T-cell lymphoma and diffuse large B-cell lymphoma	↓ Cancer tissues ↓ Cell lines		↓ Cell proliferation ↑ Apoptosis ⊣ IGF-1R/AKT/ERK1/2	Zhou et al., 2017a,b
				

## The Role of αKlotho in Cancer

### Breast Cancer

In 2008, KL was revealed as a tumor suppressor in breast cancer (Wolf et al., 2008). According to this study, normal breast tissue exhibits higher KL expression than ductal carcinoma *in situ* or invasive ductal carcinoma. Also, in less-differentiated breast cancer cell lines, *KL* expression is lower than in the non-tumor breast cell line MCF-12A or in well-differentiated MCF-7 breast cancer cells. KL overexpression reduces, whereas RNAi-mediated KL down-regulation enhances breast cancer cell proliferation. *KL* overexpression activates the FGF pathway, whereas *KL* overexpression and sKL attenuate IGF-1R activation and its downstream targets AKT1, GSK-3β, and ERK1/2 (Wolf et al., 2008). *In vitro* and *ex vivo*, methylation of the *KL* promoter in breast cancer cells is negatively correlated with *KL* mRNA abundance, suggesting a role of epigenetic silencing of KL in breast cancer (Rubinek et al., 2012; Dallol et al., 2015). Also dietary methyltransferase inhibition with green tea polyphenols and histone deacetylase inhibition with sulforaphane up-regulate epigenetically silenced KL in breast cancer cells (Sinha et al., 2015). sKL may exert further antitumor effects in breast cancer by regulating endoplasmic reticulum (ER) Ca^2+^ storage, as well as inner mitochondrial membrane potential and Ca^2+^ transport (Shmulevich et al., 2020). Heterozygosity for a certain *KL* gene variant (KL-VS) is associated with an even higher breast cancer risk of patients with *BRCA1* mutation prone to developing breast cancer (Wolf et al., 2010).

### Colorectal Cancer

Epigenetic silencing through *KL* promoter hypermethylation is observed in different colon cancer cell lines (Pan et al., 2011). Also, in human colorectal cancer (CRC) specimens, *KL* promoter methylation with reduced *KL* mRNA is frequent (Gan et al., 2011; Pan et al., 2011; Li et al., 2014; Yang et al., 2014; Perveez et al., 2015; Arbel Rubinstein et al., 2019; Liu et al., 2019; Son et al., 2020). According to some studies, methylation status and reduced *KL* expression are independent of age, gender, TNM stage, histological grade, or tumor differentiation (Pan et al., 2011; Yang et al., 2014; Perveez et al., 2015). Others found an association of KL expression with decreased survival of CRC patients (Liu et al., 2019) or TNM stage, invasiveness, and lymph node metastasis (Li et al., 2016; Arbel Rubinstein et al., 2019). Moreover, a recent study observed an association between *KL* variants and an increased risk of CRC (Kamal et al., 2020). Overexpression of *KL* or *KL1* fragment or treatment with sKL decreases surviving colonies and cell proliferation and induces cell cycle arrest and apoptosis of colon cancer cells (Pan et al., 2011; Arbel Rubinstein et al., 2019). Mice colon cancer cells transfected with KL exhibit lower tumor growth, weight, and volume (Li et al., 2014). The same holds true after treatment with sKL1 (Arbel Rubinstein et al., 2019). Similar to breast cancer, KL might be tumor-suppressing by inhibiting IGF-1R–dependent PI3K/AKT signaling (Li et al., 2014) or aerobic glycolysis via ERK/hypoxia-inducible factor 1α (HIF-1α) (Li et al., 2018) in CRC. Also, down-regulation of Wnt3a/β-catenin signaling and apoptosis are induced by KL in CRC cells (Bordonaro and Lazarova, 2015; Arbel Rubinstein et al., 2019; Xie et al., 2020). *miR-15b* may contribute to reduced KL expression in CRC because higher *miR-15b* levels in CRC patients compared to healthy subjects, those with metastasis than without, and those with cancer recurrence than without are described (Li et al., 2016). In CRC cells, inflammation-inherent nuclear factor κB (NF-κB) and IGF-1R activity further lowers KL expression, increasing cell proliferation and invasion (Xie et al., 2019). Conversely, KL blocks NF-κB activation (Liu et al., 2019).

### Lung Cancer

KL is down-regulated in lung cancer cells and tissues and even more so in chemotherapy-resistant lung cancer (Chen et al., 2012, 2016; Chen B. et al., 2018). KL inhibits lung cancer cell proliferation, growth, invasiveness, and migration and fosters apoptosis (Chen et al., 2010, 2012, 2016, 2019; Wang X. et al., 2011; Wang et al., 2013), effects, at least in part, dependent on IGF-1R/AKT (Chen et al., 2010; Wang et al., 2013) and Wnt3a/β-catenin signaling (Chen et al., 2012, 2019) and on reduced interleukin 6 (IL-6) and IL-8 production (Chen B. et al., 2018). *MiR-10b* lowers, Ras-related GTPase Ras8 up-regulates KL expression in non–small-cell lung cancer cells (Huang et al., 2015). Patients with large-cell neuroendocrine lung carcinoma or small-cell lung cancer with KL expression have better outcome than those without KL expression pointing to KL being a potential biomarker (Usuda et al., 2011a; Vanoirbeek et al., 2011; Brominska et al., 2019). This could not be confirmed for sKL in lung cancer (Pako et al., 2020). KL may sensitize lung cancer cells to apoptosis induction by cisplatin via PI3K/AKT signaling (Wang et al., 2013) or due to decreased autophagy (Chen et al., 2016).

### Hepatocellular Cancer

HCC cells and HCC tissue exhibit reduced KL expression (Shu et al., 2013; Xie et al., 2013b; Sun et al., 2015; Tang et al., 2016b), a phenomenon again explained by epigenetic silencing of the *KL* promoter through hypermethylation and acetylation (Xie et al., 2013b). *KL* promoter methylation is associated with a poorer prognosis (Xie et al., 2013b), whereas KL expression is inversely related to histological grade and clinical stage in HCC (Tang et al., 2016b). *KL* overexpression or treatment with recombinant KL or sKL decreases colony formation, cell proliferation, migration, and tumor invasion while inducing apoptosis and autophagy through inhibition of Wnt/β-catenin (Sun et al., 2015; Tang et al., 2016b) and IGF-1R/AKT/ERK signaling (Shu et al., 2013). According to another study, however, KL activates vascular endothelial growth factor receptor 2/p21-activated kinase 1, resulting in cell death resistance and favoring tumor migration and invasion (Chen et al., 2013). Thus, higher KL expression is associated with cirrhosis, venous invasion, tumor multiplicity, and a lower overall survival in HCC patients according to this study (Chen et al., 2013).

### Squamous Cell Carcinoma

Lower KL and higher DNA methyltransferase 3a (enzyme required for epigenetic alteration of *KL* promoter activity) are typical of the transition from normal tissue to oral dysplastic lesions to oral squamous cell carcinoma (SCC) (Adhikari et al., 2017). *KL* promoter methylation may predict survival prognosis in head and neck SCC with conflicting results (Alsofyani et al., 2017; Zhu et al., 2019). Higher *KL* gene expression is again associated with better survival, and *KL* methylation with gender, tumor grade, and site (Zhu et al., 2019). Survival of patients with esophageal SCC is better if the tumor expresses KL (Tang et al., 2016a). Moreover, KL expression is inversely correlated with invasion depth, histological grade, clinical stage, and lymph node metastasis in esophageal SCC (Tang et al., 2016a). In lung SCC, KL expression is associated with invasiveness (Ibi et al., 2017). KL inhibits *N*-cadherin and regulates epithelial–mesenchymal transition (EMT) (Ibi et al., 2017). Also, in cervix SCC, KL is reduced (Aviel-Ronen et al., 2016).

### Pancreatic Cancer

Pancreatic adenocarcinoma tissue or human pancreatic adenocarcinoma cell lines Panc1, MiaPaCa2, and Colo357 are characterized by reduced KL expression compared to normal pancreatic tissue (Abramovitz et al., 2011). Epigenetic silencing due to a hemimethylated *KL* promoter may account for this (Abramovitz et al., 2011). Overexpression of KL or recombinant sKL reduce survival and size of the cancer cell colonies and potentiates chemotherapeutic effects (Abramovitz et al., 2011). They inhibit IGF-1R and its downstream signaling effectors IRS-1, AKT1, and ERK1/2 as well as FGF2 pathway activation (Abramovitz et al., 2011). sKL injection also reduces tumor growth in mice (Abramovitz et al., 2011). KL expression is positively, p-IGF-1R abundance negatively, correlated with lower TNM stage and pathological grade (Jiang et al., 2014). Higher methylation of the *KL* promoter in pancreatic ductal adenocarcinoma compared to normal pancreatic tissue worsens outcome (Jiang et al., 2014). *miR-199a* lowers KL expression in pancreatic adenocarcinoma Panc1 cells (Zhang et al., 2020). KL inhibits mTOR as downstream target of AKT1 and MEK/ERK signaling in Panc1 cells (Zhang et al., 2020).

### Gastric Carcinoma

*KL* promoter hypermethylation with decreased gene expression is typical of gastric carcinomas and gastric carcinoma cell lines (Wang L. et al., 2011). KL overexpression inhibits growth and ERK1/2 activity, resulting in apoptosis of AGS and MKN28 gastric carcinoma cells (Wang L. et al., 2011). Promoter hypermethylation correlates with poorer survival of patients with gastric cancer, making it an independent prognosis factor (Wang L. et al., 2011). Restoration of KL expression reduces p-IGF-1R, p-PI3K, and p-mTOR in GC-7901 cells (Xie et al., 2013a). Similar to pancreatic cancer (Zhang et al., 2020), *miR-199a* influences KL expression in gastric cancer (He et al., 2014). The human sex determining region Y (SRY)–related high-mobility-group (HMG) box protein family member 17 (SOX17) protein also binds to the *KL* promoter in gastric cancer cells, thereby inducing KL expression (Yang et al., 2020).

### Prostate Cancer

A KL single-nucleotide polymorphism (*rs3752472*) is associated with the risk of prostate cancer (odds ratio = 1.85) (Kim et al., 2014b). Methylation in the *KL* CpG island region KL-M3, including −593 to −406 bp, accounts for the down-regulation of *KL* mRNA in prostate cancer cell lines DU145 and PC-3 (Seo et al., 2017). The same region is unmethylated in 22Rv1 prostate cancer cells exhibiting *KL* mRNA expression (Seo et al., 2017). The *KL* promoter in 22Rv1 cells is hypomethylated, and in DU145 and PC-3 cells hypermethylated (Seo et al., 2017).

### Renal Cell Carcinoma

In renal cell carcinoma (RCC) tissue and cell lines, KL protein and mRNA expression are reduced (Zhu et al., 2013; Gigante et al., 2015; Kim et al., 2016; Dehghani et al., 2018). KL expression is negatively associated with TNM stage, tumor size, shorter overall and progression-free survival (Zhu et al., 2013; Gigante et al., 2015). *KL* overexpression in RCC cells down-regulates PI3K/AKT/GSK3-β/Snail signaling, thereby inhibiting cell migration, invasion, and EMT (Zhu et al., 2013). Moreover, KL inhibits epidermal growth factor 1–dependent p38MAPK activation and IGF-1R signaling in Caki-1 cells compromising cell motility and proliferation (Zhu et al., 2013; Kim et al., 2016; Dehghani et al., 2018).

### Ovarian Cancer

Results regarding the role of KL in ovarian cancer are controversial. According to a clinical study of 189 EOC patients, 73.5% of patients exhibit detectable KL expression. sKL is associated with high tumor grade, suboptimal tumor debulking results, disease progression [hazard ratio (HR) = 1.97], and death (HR = 2.09), possibly due to KL supporting the tumor with energy and angiogenesis (Lu et al., 2008). Others found reduced KL expression in different human EOC cell lines and specimens, as well as inhibition of proliferation of different EOC cell lines upon sKL treatment or KL overexpression (Lojkin et al., 2015; Yan et al., 2017). KL suppresses IGF-1–induced ERK 1/2 phosphorylation in OVCA-432 and SKOV-3 cells (Lojkin et al., 2015). KL expression is lower in ovarian cancer and is associated with decreased survival (Yan et al., 2017). In mice, KL-expressing A2780 tumor cells grow more slowly than KL-negative tumor cells (Yan et al., 2017). KL suppresses a tumor-associated inflammatory response in mice with ovarian cancer, thereby contributing to a more favorable outcome (Yan et al., 2017).

### Melanoma

In different melanoma cell lines, a mutually inhibitory effect of Wnt5a and KL expression is established impacting on metastasis (Camilli et al., 2011). The effect of KL on Wnt5a internalization and signaling is dependent on heparan sulfate proteoglycans (Camilli et al., 2011). Moreover, KL inhibits Wnt5a-mediated filamin A cleavage through calpain, an effect contributing to reduced motility of melanoma cell lines (Camilli et al., 2011). Melanoma cells exhibit *KL* expression, depending on the age of surrounding fibroblasts (Behera et al., 2017). Older patients’ melanoma cells show lower KL expression (Behera et al., 2017). Treatment of melanoma cells with media of aged fibroblasts results in increased Wnt5a expression and less *KL* mRNA expression, compared to incubation with media of young fibroblasts (Behera et al., 2017). KL expression in melanoma cells is enhanced by PPARγ, and KL or PPARγ agonist rosiglitazone treatment reduce melanoma growth in mice (Behera et al., 2017). HMG protein B1 (HMGB1) activates NF-κB and inhibits KL expression melanoma cell lines (Xie et al., 2016).

### Thyroid Cancer

KL overexpression and sKL induce apoptosis and compromise proliferation of thyroid cancer cell lines FTC133 and FTC238, an effect presumably dependent on stanniocalcin-1 (Dai et al., 2016). Low differentiation is paralleled by reduced KL expression in human thyroid cancer (Pawlikowski et al., 2019).

## Other Forms of Cancer

KL is a possible tumor suppressor in urothelial carcinoma of the bladder (Hori et al., 2016, 2018). KL expression in glioblastoma multiforme (GBM) correlates with outcome (Trošt et al., 2016; Peshes-Yeloz et al., 2019). sKL decreases viability of GBM cell lines, and reduced KL expression is due to epigenetic *KL* promoter methylation in these cells (Peshes-Yeloz et al., 2019). Similar epigenetic mechanisms of down-regulation of *KL* expression are effective in human specimens of invasive cervical carcinoma and cell lines (Lee et al., 2010). Secreted KL acts as a tumor suppressor in CaSki cervical carcinoma cells by inhibiting canonical Wnt signaling and *c-MYC* and *Cyclin D1* expression (Lee et al., 2010). *KL* overexpression in SiHa cells down-regulates β-catenin, *c-MYC*, and *cyclin D1* signaling, as well as EMT (Lee et al., 2010; Chang et al., 2012).

*KL* expression correlates with overall survival and is lower in dedifferentiated liposarcoma (DDLPS) than in adipose tissue (Delcroix et al., 2018). *KL*-overexpressing DDLPS blunts IGF-1–induced Ca^2+^ and ERK1/2 signaling, reducing proliferation, inducing apoptosis, and sensitizing cells to ER stress (Delcroix et al., 2018).

Also in T-cell lymphoma and diffuse large B-cell lymphoma (DLBLC), KL overexpression attenuates IGF-1R, ERK1/2 and AKT signaling (Zhou et al., 2017a,b). Moreover, in biopsies and cell lines of T-cell lymphoma and DLBLC, KL expression is reduced correlating with shorter survival. *KL* overexpression in T-cell lymphoma and DLBLC cell lines lowers proliferation and enhances apoptosis (Zhou et al., 2017a,b).

## FGF23/KL and the Cancer Microenvironment

**FIGURE 2:**
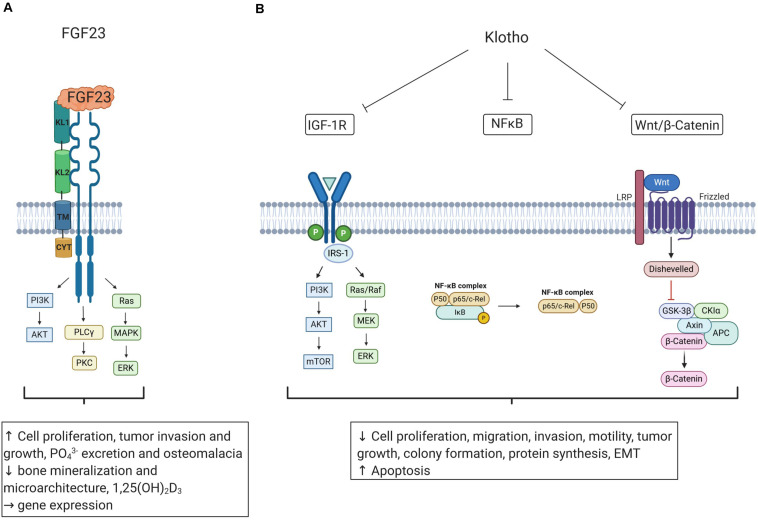
The influence of FGF23 and Klotho on oncogenic and tumor-suppressing pathways. **(A)** FGF23 binds to FGFRs and coreceptor KL and may impact cell proliferation, tumor growth, and bone gene transcription. Tumor-induced elevation of FGF23 production may cause phosphate wasting, 1,25(OH)_2_D_3_ reduction, and osteomalacia. **(B)** Klotho (KL) is a tumor-suppressor inhibiting pathways relevant for tumorigenesis including IGF-1R, Wnt/β-catenin, and NF-κB signaling, resulting in decreased cell proliferation, invasion, migration, tumor growth, protein synthesis, and EMT and inducing apoptosis. Figure according to Sachdeva et al. (2020). Created with BioRender.com. Insulin-like growth factor 1 receptor (IGF-1R); phosphoinositide 3-kinase (PI3K); mammalian target of rapamycin (mTOR); mitogen-activated protein kinase (MAPK); mitogen-activated protein kinase kinase (MEK); extracellular receptor signal-related kinase (ERK); insulin receptor substrate 1 (IRS-1); nuclear factor ‘kappa-light-chain-enhancer’ of activated B-cells (NF-κB); iκappaB kinase (IκB); low density lipoprotein receptor-related protein (LRP); glycogen synthase kinase-3β (GSK-3β); adenomatous polyposis coli (APC); phospholipase Cγ (PLCγ), protein kinase C (PKC); epithelial to mesenchymal transition (EMT); phosphate (PO_4_^3−^), 1,25(OH)_2_D_3_ (active vitamin D).

As summarized in Figure 2, KL is a potent regulator of IGF-1R and Wnt/β-catenin signaling, and these pathways are highly relevant for the cancer microenvironment (Huang and Du, 2008; Sanchez-Lopez et al., 2016). Local hypoxia is typical of advanced cancers activating HIF-1 (Petrova et al., 2018). KL inhibits HIF-1α in CRC (Li et al., 2018). Conversely, HIF-1α increases ectopic FGF23 expression in patients with TIO (Zhang et al., 2016). Hypoxia fosters accumulation of tumor-associated macrophages in the tumor microenvironment and mediates inflammation (Lewis and Murdoch, 2005). Interestingly, cultured macrophages express FGF23, which up-regulates cell number and their tumor necrosis factor α expression (Masuda et al., 2015; Han et al., 2016). Thus, FGF23 production and local inflammation may be interdependent in the microenvironment of the tumor depending on hypoxia, HIF-1α activation, and tumor-associated macrophages. Furthermore, FGF23 possibly contributes to a bone-like microenvironment in phosphaturic mesenchymal tumor, mixed connective tissue variant (PMTMCT), through FGFR1c/KL, inducing enhanced FGF23 production by the tumor cells and worsening TIO (Kinoshita et al., 2019). Clearly, further studies are warranted to address this important issue.

## FGF23/KL, Phosphate Homeostasis, and Cancer

FGF23/FGFR/KL regulate renal phosphate handling (Gattineni et al., 2009). Moreover, FGF23 indirectly impacts on phosphate by inhibiting 1,25(OH)_2_D_3_ formation (Chanakul et al., 2013) and by affecting PTH (Krajisnik et al., 2007; Kawakami et al., 2017). Hence, FGF23/KL have a central role in the interaction of bone, kidney, small intestine, and parathyroid gland, maintaining phosphate homeostasis (Razzaque, 2009b). Serum phosphate levels are higher in patients with cancer than in healthy individuals (Papaloucas et al., 2014). Higher phosphate concentrations in men are related to a higher overall cancer risk (Wulaningsih et al., 2013), and higher phosphate intake accelerates tumorigenesis in mice (Lee et al., 2015), uncovering phosphate as a possible factor in cancer (Brown and Razzaque, 2018). Accordingly, CKD patients, often exhibiting hyperphosphatemia and 1,25(OH)_2_D_3_ deficiency, have an increased risk of cancer (Wong et al., 2009, 2016; Park et al., 2019). 1,25(OH)_2_D_3_ may have anti-cancer activity (Vanoirbeek et al., 2011). According to Brown’s hypothesis, hyperphosphatemia is an important factor in tumorigenesis and at the same time causes an endocrine reduction of 1,25(OH)_2_D_3_, which in turn is associated with an increased risk of cancer (Brown, 2019). For this hypothesis, FGF23/KL plays an important role due to its pivotal function in phosphate handling. Definitely, further research on pathological derangements of phosphate homeostasis is warranted to uncover the relationship between FGF23/KL dysregulation, disturbed phosphate homeostasis, and cancer development.

## Conclusion

KL seems to be an universal tumor suppressor in many different tumor entities owing to its inhibitory effect on pro-survival intracellular pathways including IGF-1R/PI3K/AKT or Wnt signaling. Often, cell culture studies revealed similar actions of sKL and overexpression of transmembrane KL in different types of cancer. Whether targeting KL can be therapeutically exploited in cancer must be investigated in future trials. In most studies and types of cancer, higher abundance of sKL is associated with a more favorable prognosis, presumably due to its down-regulatory effect on major prosurvival signaling cascades required for cancer progression. The investigations into the role of FGF23 in cancer have so far revealed two important aspects in general: In those forms of cancer affecting bone or originating from it such as MM or prostate cancer, FGF23 signaling may directly contribute to cancer biology/progression. In many other tumor entities, the biological role of an elevation of the plasma FGF23 concentration is still enigmatic, but FGF23 may serve as a (tumor) biomarker. In TIO, treatment with anti-FGF23 monoclonal antibody offers a beneficial therapeutic intervention. In other malignancies affecting bone including prostate cancer or MM, an anti-FGF23 approach may also be useful as enhanced FGF23 or FGF23 signaling is typical of these tumor entities. Clearly, this and the role of FGF23-dependent phosphate metabolism in cancer require further studies.

## Author Contributions

All authors listed have made a substantial, direct and intellectual contribution to the work, and approved it for publication.

## Conflict of Interest

The authors declare that the research was conducted in the absence of any commercial or financial relationships that could be construed as a potential conflict of interest.

## Abbreviations

1,25(OH)_2_D_3_: active vitamin D
ADAM10: A disintegrin and metalloproteinase domain-containing proteins 10
ADAM17: A disintegrin and metalloproteinase domain-containing proteins 17
ALPL: alkaline phosphatase
BACE1: β-APP cleaving enzyme 1
CKD: chronic kidney disease
CRC: colorectal cancer
CYP: cytochrome P 450
DDLPS: dedifferentiated liposarcoma
DLBLC: diffuse large B-cell lymphoma
EGR-1: early gene response transcription factor 1
EMT: epithelial to mesenchymal transition
EOC: epithelial ovarian cancer
ER: endoplasmic reticulum
ERK1/2: extracellular receptor signal-related kinase ½
FAM20C: family with sequence similarity 20 member C
FGF: fibroblast growth factor
FGFR: fibroblast growth factor receptor
FOXO: forkhead box O
GalNT3: UDP-*N*-acetyl-alpha-D galactosamine:polypeptide *N* acetylgalactosaminyltransferase 3
GFR: glomerular filtration rate
GBM: glioblastoma multiforme
GSK-3 β: glycogen synthase kinase-3 β
HCC: hepatocellular carcinoma
HIF-1 α: hypoxia-inducible factor 1 α
HMGB1: high-mobility group protein B 1
HPSE: heparanase
HR: hazard ratio
IGF-1: insulin-like growth factor 1
IL: interleukin
KL: α Klotho
KLB: β Klotho
KLG: γ Klotho
Klph: Klotho lactase-phlorizin hydrolase-related protein
MAPK: mitogen-activated protein kinase
MDS: myelodysplastic syndromes
miR: micro ribonucleic acid
MM: multiple myeloma
mTOR: mammalian target of rapamycin
NaPiIIa: sodium phosphate cotransporter 2a
NF- κ B: nuclear factor ‘kappa-light-chain-enhancer’ of activated B-cells
PDAC: pancreatic ductal adenocarcinoma
PPAR: peroxisome proliferator–activated receptor
PI3K: phosphoinositide 3-kinase
PTH: parathyroid hormone
RCC: renal cell carcinoma
ROMK1: renal outer medullary potassium channel 1
sKL: soluble Klotho
SCC: squamous cell carcinoma
SNP: single-nucleotide polymorphism
SOX17: sex determining region Y (SRY) – related high-mobility group (HMG) box protein family member 17
TNM: tumor nodes metastasis
TIO: tumor-induced osteomalacia
TRPV5: transient receptor potential ion channel 5
VDR: vitamin D receptor
Wnt: wingless-related integration site.
